# Morgagnian Cataract

**DOI:** 10.31662/jmaj.2022-0077

**Published:** 2022-06-24

**Authors:** Takashi Ono, Kazunori Miyata

**Affiliations:** 1 Miyata Eye Hospital, Miyazaki, Japan; 2 Department of Ophthalmology, Graduate School of Medicine, The University of Tokyo, Tokyo, Japan

**Keywords:** Cataract, Ophthalmology, Eye surgery

A 63-year-old man was referred to our hospital with a complaint of decrease in the vision of his left eye. The visual acuity of his left eye was hand motion. His intraocular pressure was 18 mmHg, with no evidence of intraocular inflammation. The lens had hypermatured as a Morgagnian cataract characterized by total liquefaction of the cortex ^(1)^. This allowed the nucleus to sink (Figure 1A), as shown by transillumination (Figure 1B). Phacoemulsification and intraocular lens insertion were performed without complications. A month after the surgery, the patient’s vision improved to 1.0, allowing us to perform a detailed fundus examination, which showed no abnormality. Morgagnian cataracts are frequently reported in developing countries; however, they are rare in Japan. They can spontaneously rupture into the anterior chamber and cause severe intraocular inflammation, and early detection and treatment are required ^(2), (3)^.

**Figure 1.**
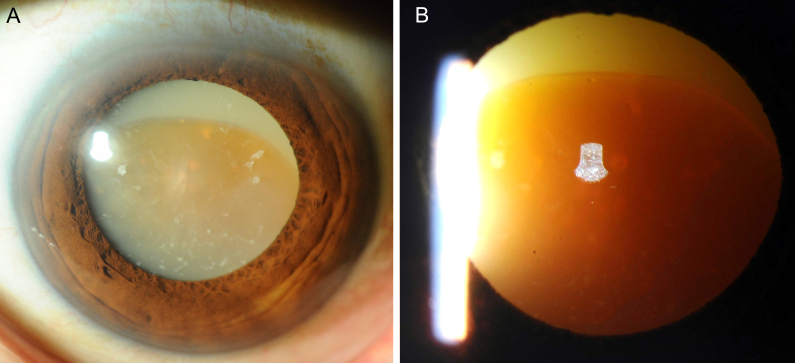
A. Image of the anterior segment obtained using slit-lamp microscopy at initial presentation. Morgagnian cataract is observed. The cortex is emulsified and the brown nucleus is sinking. B. Image of the anterior eye segment with transillumination. The solid nucleus of the lens and total liquefaction of the cortex can be observed.

## Article Information

### Conflicts of Interest

None.

### Author Contributions

T.O. and K.M. contributed to the design and conduct of the study and the collection, management, and interpretation of data. K.M. contributed to the preparation, review, and approval of the manuscript.

### Approval by Institutional Review Board (IRB)

Not applicable.

### Informed Consent

Written informed consent was obtained from the patient.
